# Research on the MEG of Depression Patients Based on Multivariate Transfer Entropy

**DOI:** 10.1155/2022/7516627

**Published:** 2022-07-20

**Authors:** Xinyu Zhang, Jicheng Xie, Changyu Fan, Jun Wang

**Affiliations:** ^1^School of Geographic and Biologic Information, Nanjing University of Posts and Telecommunications, Nanjing, China; ^2^Smart Health Big Data Analysis and Location Services Engineering Research Center of Jiangsu Province, Nanjing University of Posts and Telecommunications, Nanjing, China

## Abstract

The pathogenesis of depression is complex, and the current means of medical diagnosis is single. Patients with severe depression may even have great physical pain and suicidal tendencies. Magnetoencephalography (MEG) has the characteristics of ultrahigh spatiotemporal resolution and safety. It is a good medical means for the diagnosis of depression. In this paper, multivariate transfer entropy algorithm is used to study MEG of depression. In this paper, the subjects are divided into the same brain region and the multichannel combination between different brain regions, and the multivariate transfer entropy of patients with depression and healthy controls under different EEG signal frequency bands is calculated. Finally, the significant difference between the two groups of experimental samples is verified by the results of independent sample *t*-test. The experimental results show that for the same combination of brain channels, the multivariate transfer entropy in the depression group is generally lower than that in the healthy control group, and the difference is the best in *γ* frequency band and the largest in the frontal region.

## 1. Background Introduction

Depression is an affective disorder. Its main clinical manifestations are long-term depression, loss of interest in many things, insomnia, and even self-mutilation [1–4]. According to the data released by the World Health Organization, there are about 340 million patients with depression in the world, with a prevalence rate of 35%. There are 1 million suicidal deaths caused by mental illness every year. Depression has become the second largest disease burden after ischemic heart disease. In the suicide population, 40% of people die because of depression without timely and systematic treatment [5–7]. Therefore, how to distinguish whether patients suffer from depression is particularly important.

The development of information technology [8–11] has brought new opportunities and challenges to the diagnosis and treatment of depression [12–14]. MEG is a real-time monitoring technology used to record the brain magnetic field signal generated by neuronal activity and monitor the brain electrophysiological activity through the waveform, amplitude, and frequency of the signal [15–19]. MEG has a time resolution of millisecond, which can record neuron signals in real time without measuring brain metabolism; high spatial resolution of MEG can locate brain lesions accurately. MEG is a safe and repeatable detection method. MEG signals can penetrate the scalp without damage and invasion.

The brain can be regarded as a complex nonlinear system. As an index of nonlinear system, entropy is used to measure the complexity of nonlinear system [20, 21]. At present, the methods of sample entropy and approximate entropy have been applied to the analysis of brain magnetic and electricity data [22, 23]. On this basis, the multivariate transfer entropy used in this paper covers the dynamic characteristics and directionality, and is suitable for the complexity study of nonlinear systems. Partial information decomposition algorithm is a new algorithm for complex multivariate systems. Its main principle is to decompose the interaction of multivariate variables into four non negative and non-overlapping parts, and can separate collaborative and redundant structures. In view of the imperfect research on MEG in China, especially the research on the connectivity between patients with depression and normal brain regions stimulated by emotional pictures is relatively few. Therefore, the multivariate transfer entropy algorithm used in this paper to study the complexity and correlation of multiple channels of MEG can provide some preliminary understanding and help for the future clinical diagnosis of depression and the study of brain functional area connectivity.

## 2. The Source of Experimental Data

The MEG data of the experimental study comes from 13 groups of MEG data collected by the MEG center of the Brain Hospital Affiliated to Nanjing Medical University, including 8 groups of healthy subjects and 5 groups of patients with depression. The depression samples are patients with depression in the inpatient department of the hospital, including 3 male patients and 2 female patients. Their ages range from 18 to 35, with an average of 25. The healthy samples are from interns in the hospital of Nanjing Medical University and medical postgraduates. Their ages range from 20 to 28, with an average age of 23. All subjects have normal sensory stimulation, have no history of infectious diseases, exclude other mental diseases, do not contraindicated brain magnetic resonance examination, do not take psychiatric inhibitors recently, and sign an informed agreement with the ethics committee of Nanjing Medical University after informing the experiment and related matters.

The original data collected by MEG is .meg4 file, uses SPM8 software to adjust the corresponding time delay, converts the data in the −200 ms–600 ms time interval into MATLAB for calculation and processing, and finally corrects the baseline of the processed data and reduces the frequency. The processed data structure is the data dimension.

## 3. Introduction of Multivariate Transfer Entropy Algorithm and Frequency Division

### 3.1. Multivariate Transfer Entropy Algorithm

#### 3.1.1. Symbolization of Original Sequence

Symbolic dynamics is a complex and abstract mathematical theory to study symbolic dynamical systems. In symbolic dynamics system, the state of the system can be expressed as an infinite sequence of finite abstract symbols. In the middle and late 1960s, due to the increasingly active research on chaos, it began to become a powerful tool for analyzing various complex sequences. In the mid-1990s, people began to analyze symbolic time series. Symbolic time series is a new way to analyze information. It includes symbolic dynamics theory, chaotic time series analysis, and information theory, which makes the signal analysis simple, fast, and effective.

Symbolization mainly adopts the means of “coarse graining” to transform the original time series into the specified symbol series according to certain mapping rules. The symbolic time series can remove a large amount of redundant information on the basis of retaining the characteristics of nonlinear dynamics. Then, the key information is obtained by studying the dynamic characteristics of the symbol sequence.

The symbolic algorithm used in this experiment is the static scale symbolic method in symbolic dynamics. For the study of static symbolization, Wessel and other people proposed the time series four symbol static scale symbol method in the study of heart rate in patients with chronic heart failure.

The mapping rules are as follows:

(1) $$\begin{array}{c} s_{i}\left(x_{i}\right)=\left\{\begin{array}{c} 0:u_{1}<xi\leq \left(1+a\right)u_{1}\text{ or }\left(1+a\right)u_{2}\leq xi<u_{2},\\ 1:\left(1+a\right)u_{1}<xi<\infty \text{ or }-\infty <xi<\left(1+a\right)u_{2},\\ 2:\left(1-a\right)u_{1}\leq xi<u_{1}\text{ or }u_{2}<xi\leq \left(1-a\right)u_{2},\\ 3:\left(1-a\right)u_{2}<xi<\left(1-a\right)u_{1}. \end{array}\right. \end{array}$$

1 ≤ *i* ≤ *N* represents the symbol position corresponding to the symbolic original time series, *u*_1_, *u*_2_ is the average of the sampled signals greater than or equal to zero and less than zero in the original sequence. *a* is a special constant with a normal value range of [0.03, 0.07], because it will lose details if it is too large, and it will aggravate the influence of noise if it is too small, so it cannot better capture the dynamic information in the signal. In order that the processed signal will not lose the dynamic characteristics of the time series, it is usually taken as *a*=0.05.

#### 3.1.2. Multivariate Transfer Entropy

At present, nonlinear dynamics method [24, 25] has been widely used in brain science research. For the study of EEG signals, the nonlinear dynamic methods mainly include correlation dimension, Lyapunov exponent, Kolmogonov entropy, approximate entropy, and sample entropy. However, there are few studies on nonlinear dynamic methods of MEG signals at home and abroad. At present, the relevant nonlinear dynamic methods used in MEG research mainly include Lempel Ziv, correlation dimension, and entropy statistics.

Human brain is a complex nonlinear system, and its left and right hemispheres are asymmetric. The transfer entropy can obtain the dynamic characteristics of the nonlinear system and its direction. Therefore, the study of asymmetric entropy transfer algorithm can just meet the characteristics of brain. The traditional transfer entropy algorithm is only aimed at the research of binary connectivity, that is, the connectivity between two variables. For the MEG signals of depression, the signals of MEG channels in the same functional area and between different functional areas may affect each other.

**Figure 1:**
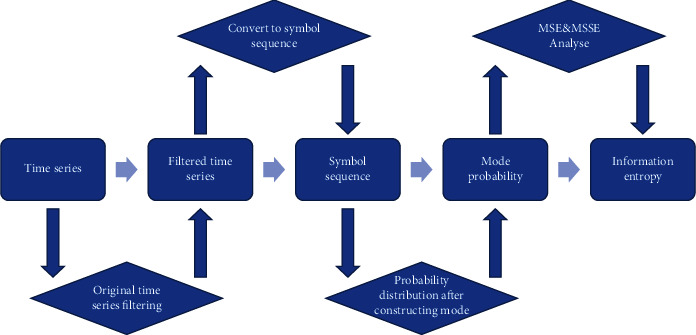
Algorithm flowchart.

Therefore, the experiment in this paper takes multivariate transfer entropy as the measurement value, which can study the coupling between multiple variables. Compared with the traditional transfer entropy algorithm, the analysis of the experimental results is more accurate. Because the calculation process is sensitive to noise and requires high parameters, the multivariate transfer entropy is generally calculated after symbolic processing of the corresponding original time series, the specific algorithm processing procedures is shown in Figure 1.

The definition of multivariate transfer entropy can be given by multivariable transfer entropy and the symbolization of the original sequence, that is, the *X* sequence is mapped into a symbolic sequence according to the symbolization rules, *S*={*s*_1_, *s*_2_,…, *s*_*i*_,…, *s*_*n*_. Sequence *Y* is mapped to a sequence of symbols, *J*={*j*_1_, *j*_2_,…, *j*_*i*_,…, *j*_*n*_, *j*_*i*_ ∈ *A*(*A*=0,1,2,3). Sequence *Z* is mapped to the corresponding symbolic order, *K*={*k*_1_, *k*_2_,…, *k*_*i*_,…, *k*_*n*_. *k*_*i*_ ∈ *A*(*A*=0,1,2,3); multivariate transfer entropy is defined asIS→JTE≈IS→JDT  E=∑psn−τ,jn,sn−τ−1,jn−τ+1,jn−τ,jn−τ−1,k,n−τkn−τ−1·,logpsn−τ,jn,sn−τ−1,jn−τ+1,jn−τ,jn−τ−1,k,n−τkn−τ−1/pjn,sn−τ−1,jn−τ+1,jn−τ,jn−τ−1,k,n−τkn−τ−1psn−τ−1,jn−τ+1,jn−τ,jn−τ−1,k,n−τkn−τ−1/psn−τ,sn−τ−1,jn−τ+1,jn−τ,jn−τ−1,k,n−τkn−τ−1.

### 3.2. The Filtering of EEG Band

**Figure 2:**
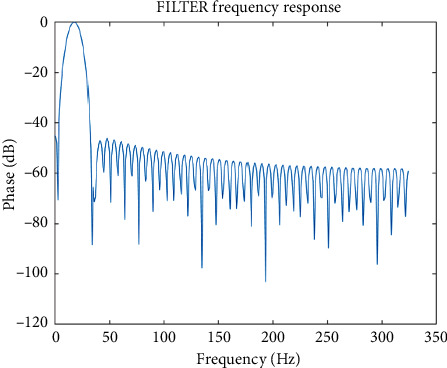
Filter frequency response.

**Figure 3:**
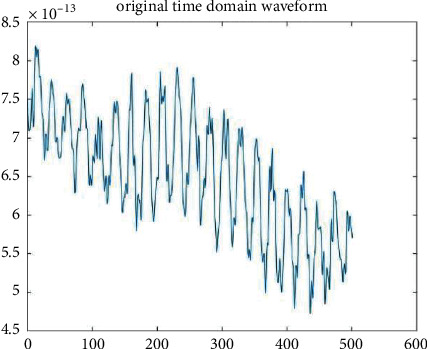
Time domain diagram of original EEG signal.

**Figure 4:**
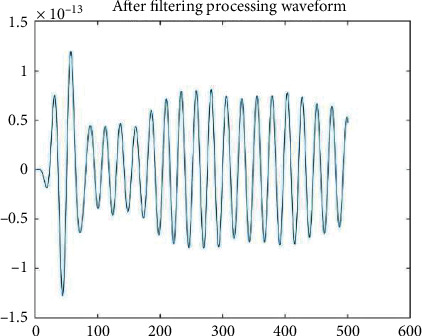
Time domain diagram of filtered b-band EEG signal.

The filter is mainly used to process signals, filter out the signals we do not need, and leave the required signal information. Generally, filters are divided into low-pass filter, band-pass filter, band stop filter, and high pass filter according to frequency characteristics. According to the difference of impulse response, the digital filter is divided into finite impulse response (FIR) filter and infinite impulse response (IIR) filter. FIR filter is an all zero structure, so the system is very stable and has the characteristics of linear phase. All signals in the effective frequency range will not have phase error. An FIR filter with band pass of 14–30 Hz is designed on the MATLAB platform, which can filter the EEG signal to obtain segment *a* in the EEG signal. The frequency response of the filter is as follows:

Figure 2 is the frequency response function of the band-pass filter. The allowable frequency is 1430 Hz, which is cut off in other frequencies.  Figure 3 is the time domain waveform of the unprocessed EEG signal, and Figure 4 is the time domain diagram of EEG signal filtered on the band. It can be seen that the fluctuation of the signal is chaotic. After the original signal is transformed from the time domain to the frequency domain, 1430 Hz is extracted, and then transformed to frequency domain to obtain *β* EEG signal. It can be seen that the waveform of *β* signal is obviously different from the original data. After that, the EEG time domain diagram under B (<4 Hz, delta), (4∼7 Hz, theta), (8∼13 Hz, alpha), and C (>30 Hz, gamma) can be obtained by using a similar method.

## 4. Frequency Division Results and Analysis of Symmetrical and Asymmetric Brain Regions

### 4.1. Verifying the Difference with Relative Error

In order to study the differences between patients with depression and healthy subjects in different brain regions, this characteristic parameter is defined to characterize the relative difference of the mean value of multivariate transfer entropy between the two groups of experimental samples.

(2) $$\begin{array}{c} \sigma =\frac{{\text{MsteEn}}_{p}-{\text{MsteEn}}_{h}}{{\text{MsteEn}}_{h}}. \end{array}$$

MsteEn_*p*_ represents the mean value of multivariate transfer entropy of patients with depression corresponding to brain channel combination (including symmetric channel combination and asymmetric channel combination). MsteEn_*h*_ represents the mean value of multivariate transfer entropy of the corresponding brain region channel combination (including symmetric channel combination and asymmetric channel combination) of healthy control group. *σ* represents the change range of multivariate transfer entropy of brain channel magnetoencephalogram signal in depression group relative to the healthy control group. The greater the absolute value of *σ*, the more obvious the difference between them; *σ* < 0 represents that the multivariate transfer entropy of patients with depression is smaller than that of normal subjects; *σ* > 0 represents that the multivariate transfer entropy of patients with depression is greater than that of normal subjects.

#### 4.1.1. Symmetrical Brain Area Analysis

**Figure 5:**
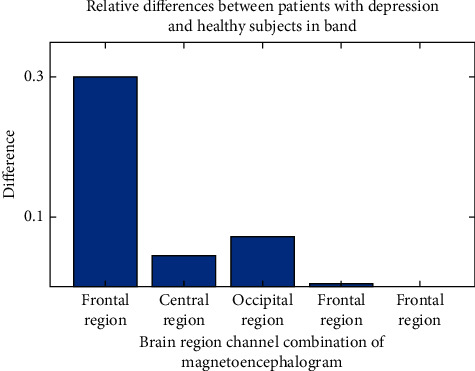
Differences of symmetrical brain regions in *θ* frequency band.

**Figure 6:**
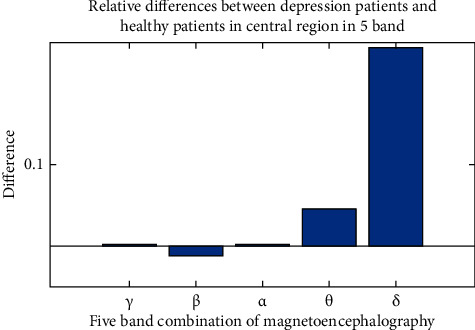
Differences of various frequency bands in the central region.

After calculating the differences of each symmetrical brain region under different frequency bands and the differences of different brain regions under each frequency band with MATLAB [26–28], we can draw a difference comparison columnar statistical chart. It is inconvenient to show all pictures due to space limitation. Take the differences of each symmetrical brain region under frequency band A and the differences of each frequency band in the central region as an example, as shown in Figures 5 and 6.

Figure 5 is the difference comparison statistical diagram of each symmetrical brain region by filtering the EEG signal to the *θ* band. It can be seen from the figure that under this band, the relative difference of frontal region is the largest, and the effect is the best to distinguish patients with depression from healthy subjects; Figure 6 is the difference comparison statistical chart of filtering EEG signals to each frequency band after selecting the central region as the study brain region. It can be seen from the figure that for this brain region, filtering the EEG signal in the *δ* band has the best effect on distinguishing patients with depression from healthy subjects; therefore, when using the multivariate transfer entropy algorithm to distinguish patients with depression from healthy subjects, if most of the EEG signals collected are in the *θ* band, the frontal region is the best; if the collected EEG signal mainly comes from the central region, the best discrimination effect is when the EEG signal is filtered in *δ* band.

#### 4.1.2. Asymmetric Brain Area Analysis

**Figure 7:**
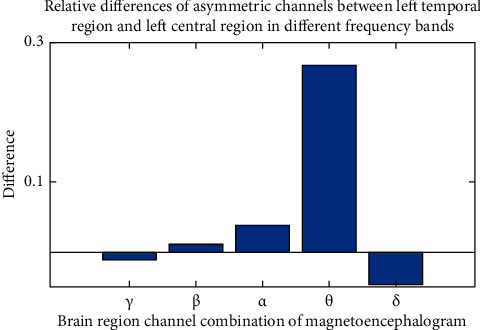
Relative difference between left frontal area and left central area under each frequency band.

**Figure 8:**
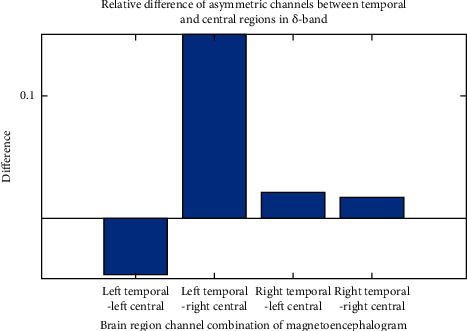
Relative difference between temporal region and central region in *δ* band.

After calculating the differences of asymmetric brain regions in different frequency bands and the differences of asymmetric brain regions in different frequency bands with MATLAB, we can draw a difference comparison columnar statistical chart. It is inconvenient to show all pictures due to space limitation, taking the multivariate transfer entropy difference between the combination of asymmetric channels from temporal region to central region under *δ* band and asymmetric channels from left temporal region to left central region under different frequency bands as an example, as shown in Figures 7 and 8.

Figure 7 represents the multivariate transfer entropy difference comparison of the asymmetric channels from the left temporal region to the left central region under different frequency bands. The connection strength difference between the brain region channel combination depression patient group and the healthy control group under each frequency band is obvious, and the discrimination relationship is *θ* > *δ* > *α* > *β* > *γ*; the multivariable sign transfer entropy difference between depression and healthy people under frequency band *θ* is greater than that under other frequency bands.  Figure 8 represents the relative difference under the asymmetric channel combination from temporal region to central region in the *δ* band. The connection strength of each brain region channel combination, depression patient group, and healthy control group is significantly different, and the differentiation relationship is left temporal region to right center > left temporal region to left center > right temporal region to left center > right temporal region to left center > right temporal region to right center. The multivariate transfer entropy of depression and healthy people under the combination of left temporal region to right central channel is more different than that under other channel combinations.

### 4.2. Improved Multivariate Transfer Entropy Analysis for Brain Magnetic Channel Signals in the Same Brain Region

The study of the four brain regions is divided into left brain region ⟶ right brain region and left brain region ⟶ right brain region connectivity analysis. In multivariate analysis, a single variable is controlled as a known condition. When analyzing the central channel of the corresponding brain region of the left and right hemispheric connectivity of the same brain region and the symmetrical and asymmetric channels of the left and right brain regions, when analyzing a single channel, the other two channels remain unchanged as a known condition, and the multivariate transfer entropy of different channel combinations is calculated. Then, do independent sample *t*-test analysis on differentiated individual subjects to verify whether the normal control group and patients with depression can be significantly distinguished in the corresponding brain regions. Secondly, with the differentiation between the four functional areas, patients with depression and healthy control group are compared. Finally, based on the calculation results of multivariate transfer entropy of the same sample under different stimuli, combined with the existing physiological basis, the changes of brain connectivity in patients with depression and healthy controls under different stimuli are analyzed.

**Figure 9:**
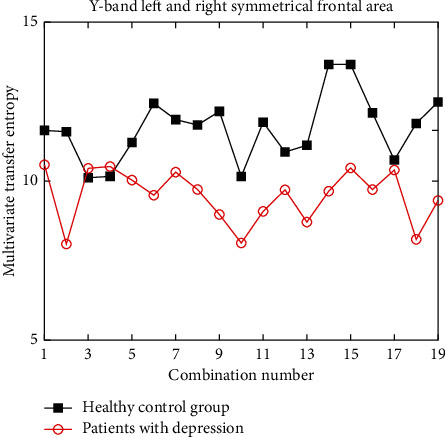
Multivariate transfer entropy of left and right symmetrical frontal region in band *γ*.

**Figure 10:**
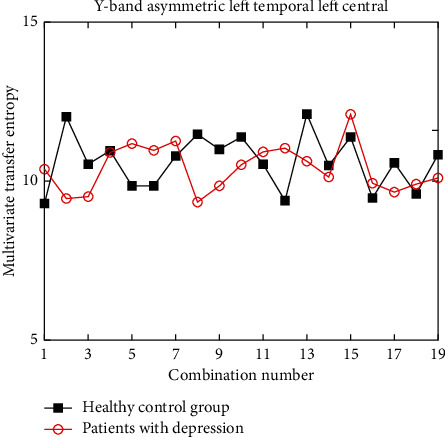
Multivariate transfer entropy of left temporal and left central region under *γ* band.

The frontal area is an important part of the brain. The frontal area mentioned in this experiment roughly corresponds to the prefrontal part of the brain and has advanced cognitive functions such as thinking ability. According to the brain asymmetry and the analysis of the same brain area, three frontal channels (left middle and right) are selected to calculate the corresponding multivariate transfer entropy. Because there are too many different combinations of the three channels, the study of simple symmetric channels is too one-sided. In order to make the above experimental conclusions more rigorous, we not only study the symmetric channels but also analyze each channel combination in the left and right frontal regions. For the frontal channel combination, the multivariate transfer entropy in the healthy control group is significantly higher than that in the depression group, but it does not rule out the existence of individual channel combinations. In order to better study the statistical significance of the sample data and study the significance of distinguishing between depression patients and healthy controls in the frontal channel, an independent sample *t*-test is performed on each channel combination data with SPSS software to verify the significance of multivariate transfer entropy algorithm in distinguishing depression patients and healthy controls. Because there are too many *t*-test results, only two groups of *t*-test results are selected here as an example. The *P* value of *t*-test results of independent samples in left and right symmetrical frontal area under *γ* band is 0.004 < 0.05, that is, there is a significant difference between depression group and healthy control group, as shown in Figures 9 and 10.

Figure 9 shows the comparison of multivariate transfer entropy in the left and right symmetrical frontal regions of patients with depression and healthy controls under band *γ*. The horizontal axis coordinate is the combination of symmetrical and asymmetric channels in the corresponding brain region, and the vertical axis coordinate is the improved multivariate transfer entropy. The black line represents the healthy control group and the red line represents the depression group. It can be seen from the figure that the multivariate transfer entropy of the healthy control group is generally higher than that of the patients with depression, and the difference is significant. Therefore, taking the *γ* band subfrontal channel as the research object can provide reference significance for the clinical diagnosis of distinguishing healthy people from patients with depression.

### 4.3. Counter Example Analysis of Multivariate Transfer Entropy of Brain Magnetic Channels in Different Brain Functional Areas

**Figure 11:**
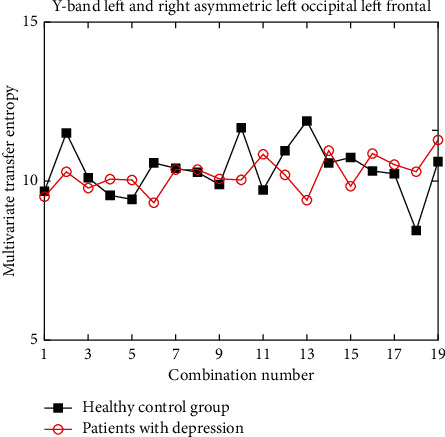
Multivariate transfer entropy of left occipital and left frontal region under *γ* band.

Under different wave bands, for the channel combinations that meet the difference in different brain functional areas, the improved multivariate transfer entropy in the healthy control group is significantly higher than that in the depression group, but it does not rule out the existence of individual channel combinations. Due to space limitations, it is not convenient to show all the example diagrams in this paper. Here, only two indistinguishable counter examples are shown, as shown in Figures 10 and 11.

The experimental results show that under the *γ* band, the discrimination between the left temporal region ⟶ the left central region, the left frontal region ⟶ the left occipital region is small, and the two are close and almost indistinguishable. Therefore, when selecting asymmetric brain regions as the research object under *γ* band, we need to carefully consider the specific brain regions selected, such as between the left temporal region and the left central region, which is difficult to provide reference significance for clinically distinguishing healthy people from patients with depression.

## 5. Summary

The results show that the multivariate transfer entropy method and frequency division brain region research proposed in this paper can well distinguish the symmetrical channel combination of various symptomatic brain regions of normal people and patients with depression. The synergistic value of multiple TE: the symmetrical meridians of frontal lobe in healthy people are significantly higher than those in patients with depression, especially when the target object is the frontal lobe of *γ* band, the difference is the most significant, which indicates that the brain activities of healthy people have better connectivity than those in patients with depression. We hope that the experimental methods provided in this paper will help to further study the difference between patients with depression and healthy people, which can provide some help for clinical diagnosis and treatment of depression.

## Data Availability

The data used to support the findings of this study are available from the corresponding author upon request.
